# Assessing Compliance With Ottawa Foot and Ankle Rules in the Emergency Department: A Closed-Loop Quality Improvement Project

**DOI:** 10.7759/cureus.84908

**Published:** 2025-05-27

**Authors:** William Wallace, Neil Limaye, Chirag Rao, Siddharth Menon, George Ratcliffe, Ahmed Mostafa, Christopher Blakeley

**Affiliations:** 1 Department of Emergency Medicine, Croydon Health Service National Health Service (NHS) Trust, London, GBR; 2 Department of Emergency Medicine, Chelsea and Westminster Hospital National Health Service (NHS) Foundation Trust, London, GBR; 3 Department of Emergency Medicine, Kingston Hospital National Health Service (NHS) Foundation Trust, London, GBR; 4 School of Medicine, King's College London, London, GBR; 5 Department of General Surgery, Lewisham and Greenwich National Health Service (NHS) Trust, London, GBR

**Keywords:** ankle injuries, ankle x-ray, foot injuries, foot x-ray, ottawa ankle rules, ottawa foot rules, quality improvement project (qip)

## Abstract

Introduction

Traumatic foot and ankle injuries are frequent presentations to the emergency department (ED). The Ottawa Foot and Ankle (F&A) Rules are to help clinicians in decision making whether an X-ray of the foot or ankle is justified.

Methods

A retrospective analysis of ankle and foot X-ray requests from a South London Hospital ED was performed. Consecutive requests for both foot and ankle X-rays were assessed chronologically until n=100 for each cohort. The clinical documentation of each ED clerking and the X-ray request clinical details were then compared to the Ottawa Rules of that respective request. The presence of a midfoot or ankle fracture was recorded. Several interventions were implemented to improve compliance; this included distributing the results of the first audit cycle and a handout containing the Ottawa F&A rules to ED staff. A teaching session for ED Junior Doctors on assessing, documenting, and requesting X-rays for foot and ankle injuries was delivered within the department. An identical second retrospective audit cycle for both ankle and foot X-rays was then completed three weeks after implementation of the above interventions.

Results

The compliance of Ottawa Foot Rules in documenting foot injuries significantly improved from 48% to 78% (p<.001). Foot X-ray request compliance significantly improved from 32% to 54% (p<.01). Documentation compliance of ankle injuries significantly improved from 74% to 87% (p<.05). Ankle injury X-ray request compliance improved from 43% to 57% (p<.05). Fracture rate did not significantly change; ankle fractures increased from 17% to 22% (p=.372), foot fracture rate remained 15% in both cycles. Significance was assessed using the Chi-square test throughout.

Conclusion

This two-cycle Quality Improvement Project demonstrated significant improvement in compliance with Ottawa F&A Rules for ED clerking documentation and X-ray request clinical details in a South London Hospital. Compliance with Ottawa F&A Rules has the potential to reduce costs, facilitate faster discharge, and reduce unnecessary radiation exposure.

## Introduction

Traumatic Foot and Ankle injuries are a common emergency department (ED) presentation, which can range from soft tissue injuries to complex fractures [1,2]. The management of these injuries can vary greatly, and diagnosis remains predominantly based on radiological imaging [1]. The Ottawa Ankle Rules (OAR) and the Ottawa Foot Rules (OFR) are decision-making tools to aid clinicians in determining whether an X-ray is indicated or not for traumatic ankle or midfoot injuries [3]. These rules are approved by the National Institute for Health and Clinical Excellence (NICE) and incorporated into their guidelines for assessing both foot and ankle injuries [4].

The OAR recommends an X-ray if there is pain in the malleolar zone along with one of the following: inability to weight bear or tenderness along the posterior edge or tip of either malleolus. The OFR, on the other hand, recommends an X-ray if there is midfoot pain along with the inability to weight bear, navicular tenderness, or tenderness at the base of the 5th metatarsal [3]. Previous studies have shown the OAR to have high sensitivity and moderate specificity. Compliance with the rules can reduce the number of unnecessary radiographs by 30-40% [5,6]. Therefore, compliance has the possibility to not only reduce unnecessary radiation exposure but also impact resource allocation and financial pressures. Previous studies tend to assess only OAR or combine the Foot and Ankle rules together for assessing compliance, sensitivity, or specificity of the rules [7]. This study was designed to assess compliance with the rules for requesting ankle and foot X-rays separately.

The Royal College of Radiologists (RCR) dictates that all radiographs should provide adequate clinical information, as per Regulation 10 (5) in The Ionising Radiation (Medical Exposure) Regulations (IR(ME)R) Guidelines (2017) [8,9]. The standard for both audit cycles is that both the ED clerking documentation along with the X-ray request clinical details should contain appropriate information regarding the respected Ottawa Rule for all cases.

The closed-loop Quality Improvement Project (QIP) aimed to assess baseline compliance with the Ottawa Foot and Ankle Rules and evaluate the impact of targeted educational interventions on improving compliance in ED documentation and imaging requests. This Quality Improvement Project (QIP) has previously been presented as an oral presentation and abstract at the Uniten Kingdom Foundation Programme (UKFPO) National Foundation Doctors Presentation Day on January 17, 2025, and as a poster presentation at RSM Tomorrow’s Doctors International Conference 2024 on July 20, 2024.

## Materials and methods

Study design

This two-cycle QIP retrospectively analysed ED clerking documentation and X-ray requests of a South London district general hospital (DGH). The initial cycle was conducted in March 2024, and the second in May-June 2024; both cycles had identical methodologies. The second cycle was initiated three weeks after interventions were implemented within the emergency department in mid-April 2024, to assess for any change in compliance. Both audit cycles were conducted with the Plan Do Study Act (PDSA) NHS Quality Improvement framework [10]. Outcomes of the project were compliant with SMART goals [11]. Cycles were registered with the local audit team with registration numbers 2024/034 and 2024/058, respectively. This QIP has been reported in accordance with Standards for QUality Improvement Reporting Excellence (SQUIRE 2.0) guidelines [12]. 

Ethical considerations

This project was performed as a QIP and was assessed using the Health Research Authority (HRA) decision tool [13]. This determined that it does not constitute research and thus, no formal NHS Research Ethics Committee approval was necessary; any ethical concerns were discussed with the project supervisor. No patient identifiable information has been included in this manuscript and therefore patient consent for publication was not required.

Participant selection

**Figure 1:**
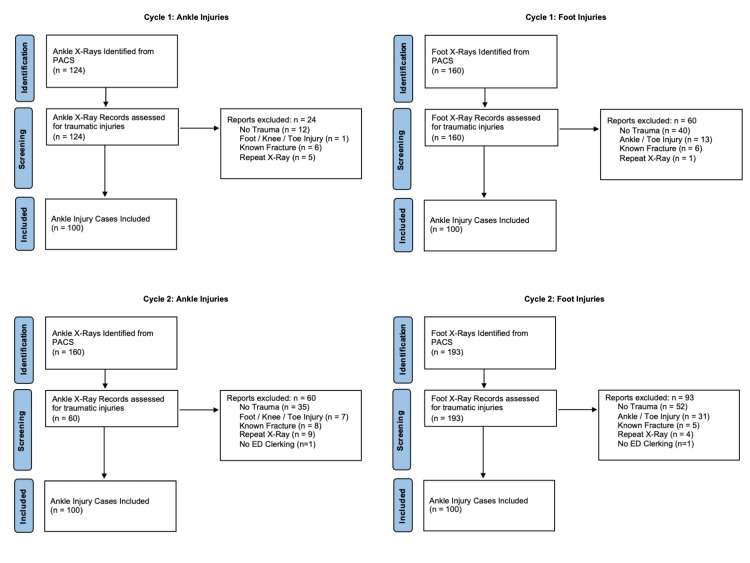
Ottawa case selection flowcarts Flow charts demonstrating case selection for Ankle and Foot injuries for both Cycle 1 and 2. Exclusion reasons have been included for each cohort.

Foot and ankle X-rays performed by the ED were collated retrospectively from the hospital’s Picture Archiving and Communication System (PACS) to identify participants for each cycle, who were subsequently anonymised. Included cases were those that had sustained an injury to the ankle or midfoot, for ankle injuries and foot injuries, respectively. Exclusion criteria for both cohorts included: atraumatic history, known fractures prior to presentation, or repeat X-rays. Those with injuries that fell into neither ankle nor midfoot injuries, for example, injuries of the phalanges, were also excluded. Cases were reviewed in a continuous and chronological order until a sample size of 100 was collated for each injury group (Figure 1). The initial radiology report for each X-ray was also reviewed, and the presence of a fracture was recorded to calculate the fracture rate. Participant selection was conducted by the same authors in both cycles to maintain consistency.

Assessing compliance

The ED clerking documentation and the X-ray request for each included case were reviewed and assessed for compliance with Ottawa Foot or Ankle Rules regarding X-ray requests. The documentation and X-ray request for a foot X-ray was deemed compliant if there was midfoot pain and one of the following was documented: inability to bear weight (walk four steps) immediately after the injury and when examined, or bone tenderness of the navicular bone or the base of the fifth metatarsal.

The documentation and X-ray request for ankle X-ray was deemed compliant if ankle pain and one of the following was documented: bone tenderness along the distal 6 cm of the posterior edge of the fibula or tip of the lateral malleolus, bone tenderness along the distal 6 cm of the posterior edge of the tibia or tip of the medial malleolus, or the inability to bear weight (walk four steps) immediately after the injury and when examined. Compliance of cases was conducted by the same authors for both cycles to maintain consistency.

Interventions

Following the initial cycle, several interventions were implemented to improve compliance; this included distributing the results of the first audit and a handout highlighting the Ottawa F&A rules to ED staff (doctors, triage nurses, and advanced care practitioners). A small group, in-person teaching session for ED Junior Doctors on assessing, documenting and requesting X-rays for foot and ankle injuries was delivered within the department. These interventions were delivered over the course of two weeks in mid-April 2024.

Data analysis

The following data were recorded for each case in Excel (Microsoft Corporation, 2024): age, gender, radiograph laterality, documentation compliance, and the presence of a fracture. GraphPad Prism 10.2.3 (GraphPad Software, CA) was used for statistical analysis and generation of figures. Pearson’s Chi-square was used to assess statistical significance. Differences were deemed significant where p<.05.

## Results

Participant data

The mean age of those receiving ankle X-rays was 32.3 (±21.3), with 51% of the sample being female in Cycle 1. Right ankle X-rays composed 62% of the ankle X-ray cohort. The mean age for the foot X-rays was 33.4 years with a standard deviation of 22.9 (Table 1). For the second cycle, shown in Table 2, the mean age was 32.2 (±21.4) for the ankle X-rays, with 64% of the cases being female. 54% of ankle X-rays were of the left ankle. The second cycle foot X-ray cohort had a mean age of 28.5 (±20.1), with 44% being male (Table 1). 

**Table 1:** Participant data for Cycle 1 and Cycle 2 Participant data for Cycle 1 and Cycle 2. The data has been distributed into population demographics and the imaging request for both Ankle Injuries (n=100) and Foot Injuries (n=100) for each cycle.

Cycle 1 Participant Data		
Ankle Injuries (n=100)	Characteristic	Frequency
Population Demographics	Age (±SD)	32.3 (± 21.3)
	Male	49
	Female	51
Imaging Request	Ankle Left	38
	Ankle Right	62
	Ankle Both	0
Foot Injuries (n=100)	Characteristic	Frequency
Population Demographics	Age (±SD)	33.4 (± 22.9)
	Male	51
	Female	49
Imaging Request	Foot Left	34
	Foot Right	65
	Foot Both	1
Cycle 2 Participant Data		
Ankle Injuries (n=100)	Characteristic	Frequency
Population Demographics	Age (±SD)	32.2 (± 21.4)
	Male	36
	Female	64
Imaging Request	Ankle Left	54
	Ankle Right	46
	Ankle Both	0
Foot Injuries (n=100)	Characteristic	Frequency
Population Demographics	Age (±SD)	28.5 (± 20.1)
	Male	44
	Female	56
Imaging Request	Foot Left	55
	Foot Right	44
	Foot Both	1

Compliance with the Ottawa Ankle Rules for ankle injuries

**Figure 2:**
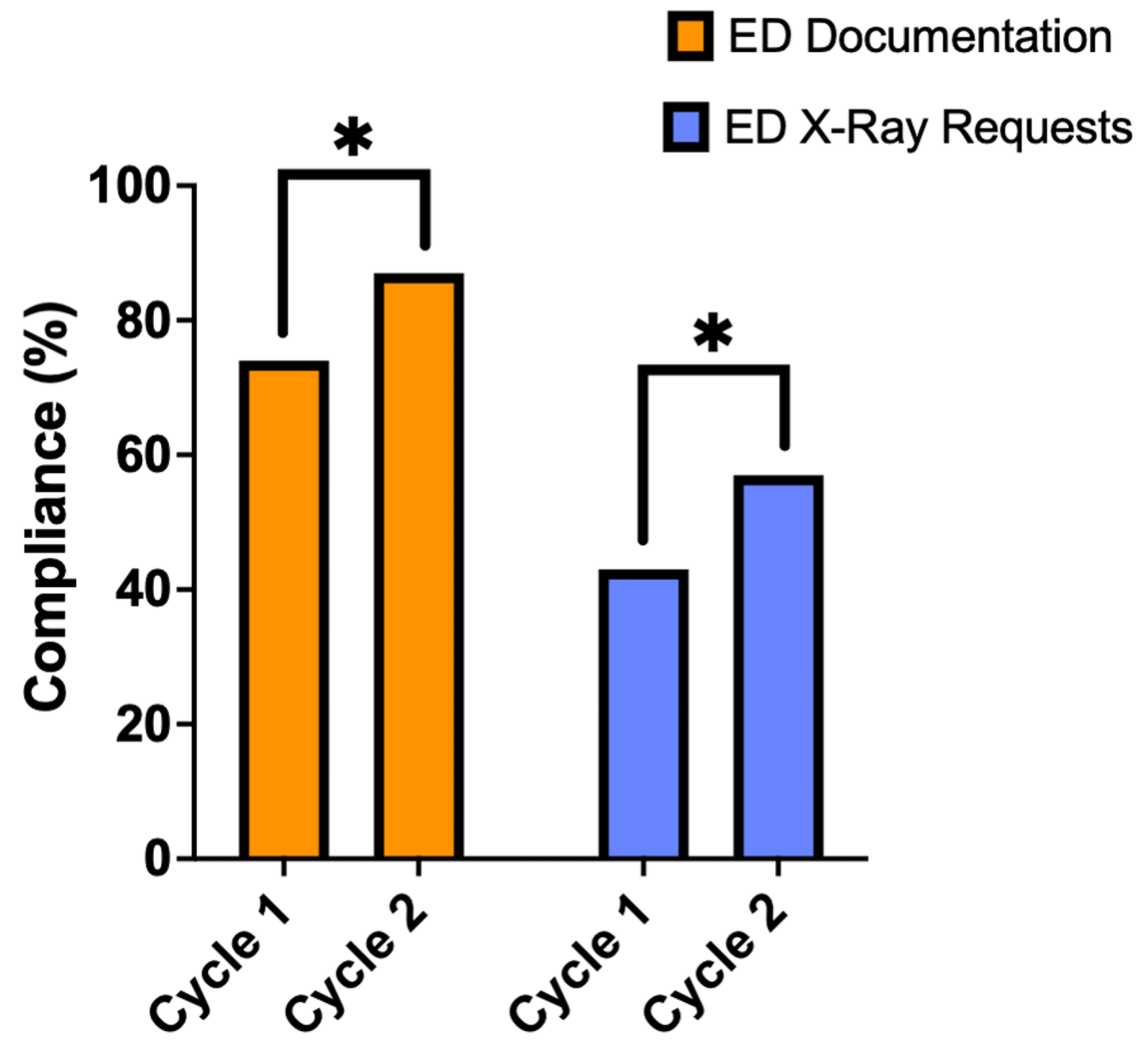
ED documentation and X-ray request compliance for ankle injuries Column graph demonstrating emergency department (ED) documentation compliance (Orange) and X-ray request compliance (Blue) for ankle injuries (n=100) for both Cycle 1 and Cycle 2. A Chi-square test was used to assess for significant differences between cycles. Significance demonstrated with asterisks: p<.05 (*), p<.01 (**), p<.001 (***) and p<.0001 (****).

Documentation compliance with OAR in Cycle 1 was 74%, which increased significantly to 87% in Cycle 2 (p <.05) (Figure 2). X-ray request compliance also significantly increased from 43% to 57% after the interventions were implemented (p<.05). 

Compliance with the Ottawa Foot Rules for foot injuries

**Figure 3:**
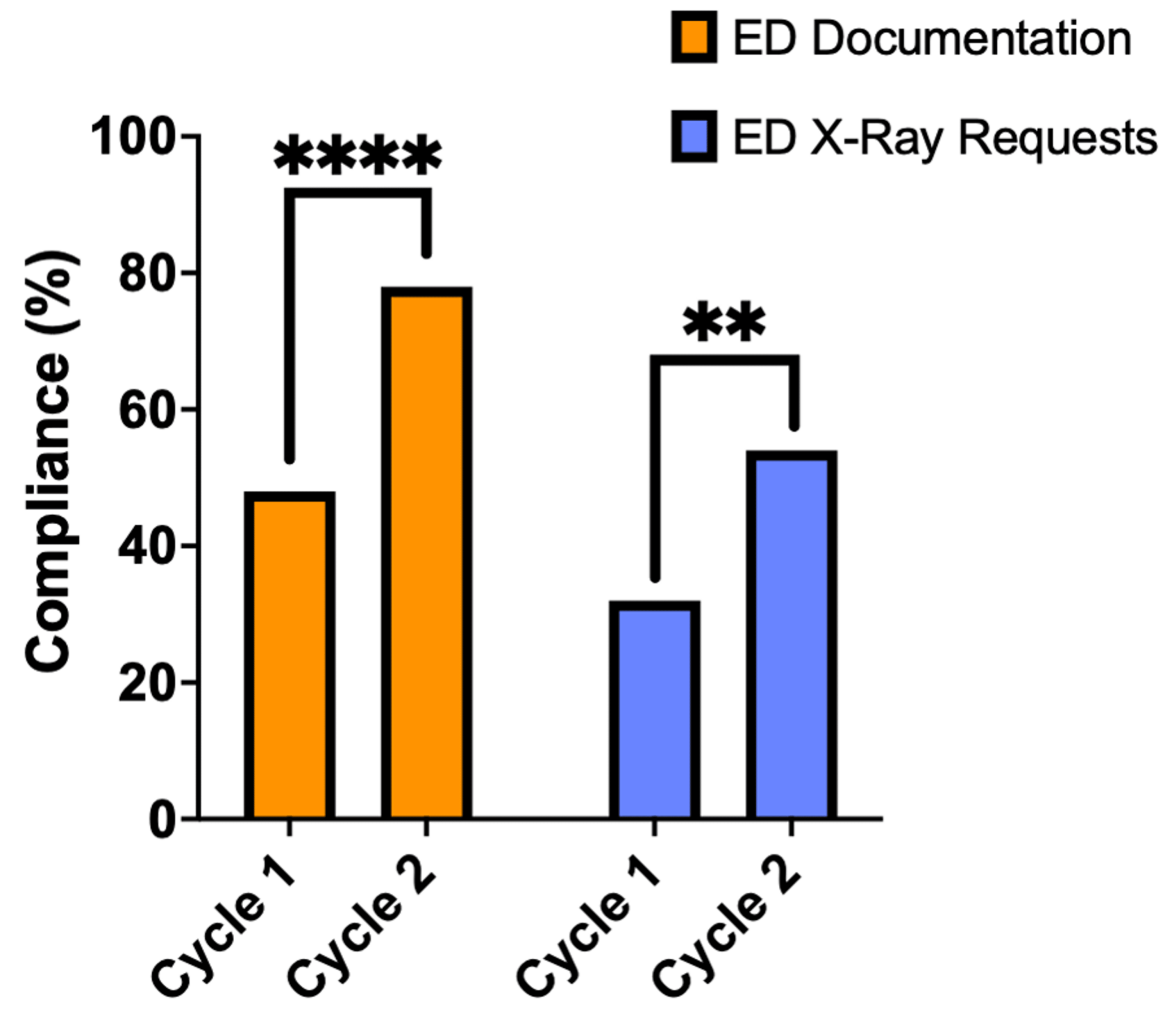
ED documentation and X-ray request compliance for foot injuries Column graph demonstrating emergency department (ED) documentation compliance (Orange) and X-ray request compliance (Blue) for foot injuries (n=100) for both Cycle 1 and Cycle 2. A Chi-square test was used to assess for significant differences between cycles. Significance demonstrated with asterisks: p<.05 (*), p<.01 (**), p<.001 (***) and p<.0001 (****).

Compliance with OFR followed a similar trend, significantly increasing from 48% to 78% for ED clerking documentation after interventions were introduced, p<.0001 (Figure 3). The X-ray request compliance increased from 32% to 54% for Foot X-rays, p<.01 (Figure 3).

Foot and ankle fracture rates

**Figure 4:**
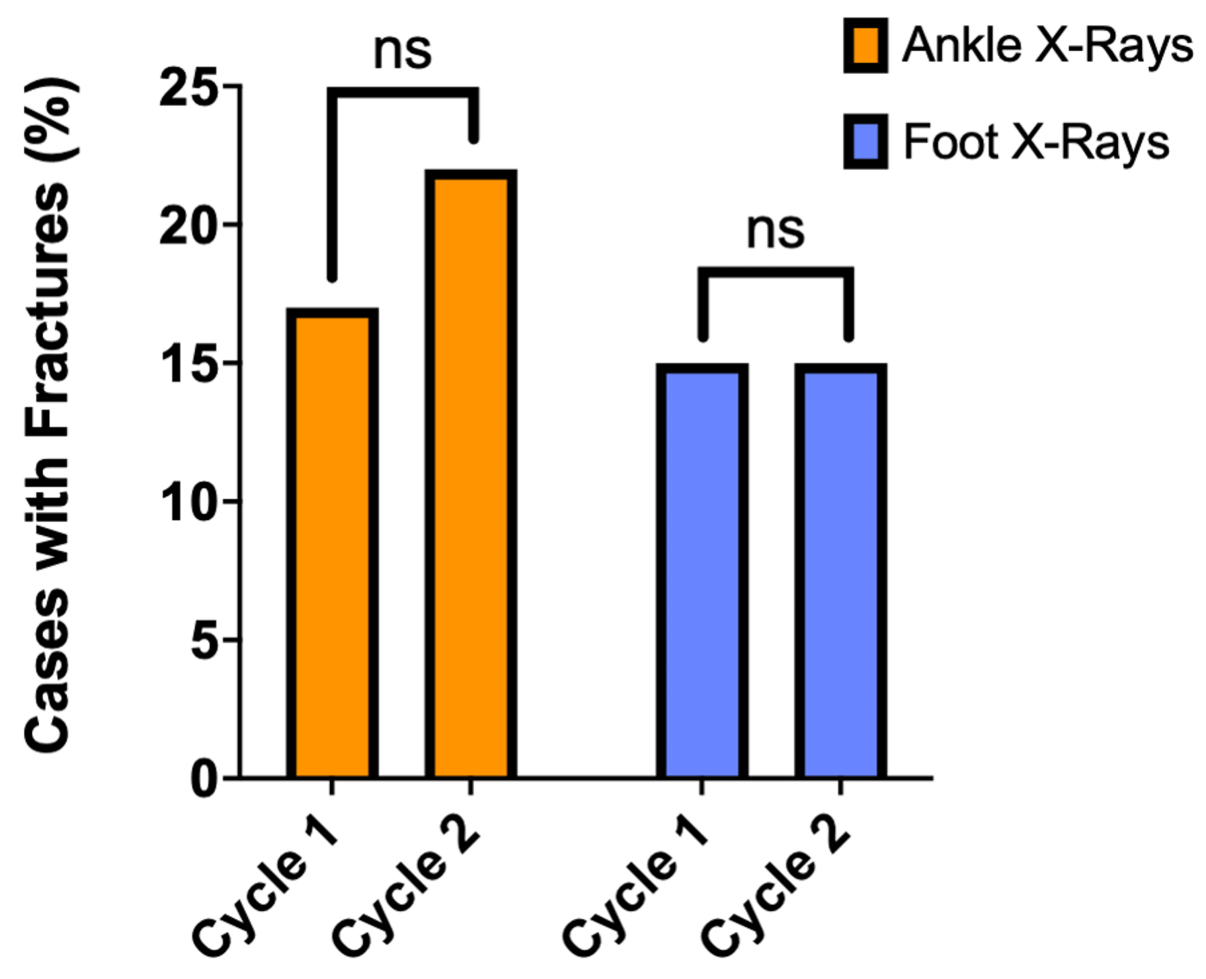
Ankle and midfoot fracture rates Column graph demonstrating the ankle fracture rate (%) of ankle Injuries (n=100, Orange) and the midfoot fracture rate of foot Injuries (n=100, Blue) for both Cycles 1 and 2. The Chi-square test was used to assess for significant differences between cycles. Non-statistically significant difference denoted with ns (p>.05).

The ankle fracture rate increased from 17% to 22%, but was not statistically significant (p=.37) (Figure 4). The rate of midfoot fractures in foot X-rays was 15% in both cycles.

## Discussion

Key findings

This closed-loop QIP assessed ED documentation and X-ray request compliance with Ottawa Foot and Ankle Rules and demonstrated statistically significant improvement following the implementation of a range of both active (teaching session on OFR and OAR for ED Junior/Resident Doctors) and passive (poster handout and mass email) interventions. 

QIP impact on documentation, X-ray request compliance and fracture rate

The ED documentation compliance for ankle injuries significantly improved from 74% to 87% (p<.05) and from 48% to 78% (p<.0001) for foot injuries. X-ray request compliance significantly improved from 43% to 57% (p<.05) and from 32% to 54% (p<.01) for ankle injuries and foot injuries, respectively. The documentation and X-ray request compliance remained lower in both cycles for foot injuries compared to ankle injuries. This could suggest that the clinicians had greater awareness of the OAR compared to the OFR; this explanation is further strengthened by the greater increase in compliance of OFR post-interventions. 

Fracture rate did not significantly change between cycles for either foot or ankle injuries. The midfoot fracture rate remained at 15% in both cycles, and the ankle fracture rate increased from 17% to 22%. The fracture rate of those with an X-ray for a midfoot or ankle injury is typically less than 15% [5]. The increase in ankle fracture rate seen could suggest a reduction in the number of unnecessary X-ray requests in the second cycle, supported by the increased documentation compliance for ankle injuries, although the difference was not statistically significant. 

Current literature typically assesses the sensitivity or specificity of the OFR, OAR, or both rules; however, there is relatively limited recent literature assessing the level of compliance of EDs with the rules [7]. A previous audit published in 2024 assessed compliance of the ED for Ottawa Ankle and Ottawa Knee Rules, which have some similarities and differences to this QIP [14]. The authors found a compliance of 33.1%, which increased to 75.8% in their second cycle, and the fracture rate increased from 32.0% to 44.0% post-intervention. Key differences between the study by Ali et al. and this QIP include the assessment of OFR, X-ray request details, and equally sized sample sizes for statistical analysis [14]. However, both studies highlight the impact that relatively simple interventions can have on compliance with the Ottawa Ankle Rules within the ED.

Importance of compliance with Ottawa Foot and Ankle Rules

There were over 300,000 sprained ankles and over 100,000 sprained foot ED diagnoses in England for the 2023/24 financial year [2]. A plain radiograph is estimated to have a relative unit cost to the NHS of £56, whilst the individual cost is relatively low; the cumulative financial cost on the NHS is considerable [15]. The rate of unnecessary radiographs decreases by 30-40% when the Ottawa Ankle Rules are used [5,6]. This demonstrates the scale of the importance of maintaining compliance with the Ottawa Foot and Ankle Rules and the potential financial benefits for NHS Trusts. Interestingly, the Ottawa Foot and Ankle Rules were initially created in 1992 to create a cost-effective method of assessing traumatic foot and ankle injuries, and these rules still prove useful over 30 years later [3]. 

ED attendances in the UK are ever-increasing [16]. The 2024 Darzi report highlighted the current state of the NHS and the burden it is under, one aspect being that EDs are more frequently failing their four-hour time limit, estimated to be 40% of patients in May 2024 [17]. Given that musculoskeletal problems make up a significant cohort of ED attendances, optimising efficiency in this area may prove beneficial [2]. Reducing the number of unnecessary radiographs could help reduce the use of resources in a hospital and the burden on radiology departments; it has the potential to reduce waiting times for those needing radiographs and enable faster discharge for those who do not require them [1]. Managing sustainable value in healthcare is imperative, and this project focused on ensuring patients with higher risk injuries were imaged as a priority, balancing patient care and finite resources [18].

The IRMER guidelines exist to maintain patient safety, avoid unwarranted radiation exposure, and keep any exposure As Low As Reasonably Possible (ALARP) [9]. The radiation dose of a limb radiograph is less than 0.001 mSv, which equates to a similar amount of background radiation exposure over three hours [19]. Whilst it could be considered that this is negligible, the argument still stands that healthcare professionals should avoid unnecessary radiation exposure for their patients where possible. Adhering to Ottawa Foot and Ankle Rules would reduce the number of unnecessary radiographs and thus reduce radiation exposure to patients [20].

While improving documentation and X-ray request details and ensuring that they both give details of how the presentation meets Ottawa Rules would help improve compliance, there are other barriers that can exist at an individual or organisational level. A main barrier is the lack of an individual’s confidence in clinical ability to exclude a fracture; thus, it highlights the potential need for education in this area of clinical medicine [21]. Other barriers included concerns about missing a fracture and its potential repercussions, as well as issues within the organisation. For example, triage nurses may request the radiograph prior to the patient being clinically assessed solely based on the history [21]. Local audits on current ED compliance and identifying potential local-specific barriers may improve compliance further. 

QIP limitations and future work 

Limitations were identified with this QIP that could be improved in further work. NICE guidelines state that Ottawa Foot and Ankle rules should be used to determine if an X-ray is warranted in those over the age of five; however, there may be an unconscious bias for a lower threshold to X-ray children to avoid a missed fracture [21-23]. The difference between compliance in children and adults would be an interesting avenue to explore further. The total sample size of each cohort was 100 for each cycle; this gives a valid snapshot of current compliance with OAR and OFR; however, it limits the ability to conduct detailed subgroup analysis in different age groups, which would have impacted the validity of statistical conclusions. The use of the initial X-ray report to assess for fracture presence maintained a constant standard for assessing fracture rate; however, there is a possibility of missed fractures, which would impact the results, although the impact of this is likely small given the fracture rate consistent with pre-existing literature [5]. Going forward, larger national and regional studies could inform practice on a greater scale, with longer periods of time for data collection. 

The retrospective cross-sectional nature of this project means that although a significant difference in compliance was seen between cycles, we cannot confirm causality. This study design also has limitations, including potential selection or observer bias, although these were limited with a fixed selection criterion and a defined measure of compliance, as well as the same reviewers for both cycles. One of the interventions was to highlight current standards within the department, whilst this was aimed to highlight current compliance as well as educate people about the Ottawa Foot and Ankle Rules; there is possibility of clinician’s actions changing as they were then aware that they could be audited, however this confounder cannot be avoided. Despite multiple interventions being implemented and a significant increase in compliance measured, there is a limitation to long-term improvement. No long-standing intervention implemented, and given the rotational nature of Junior/Resident Doctor jobs, improved compliance rates may not be sustained. Due to this, an improvement in the evaporation effect may occur, leading to compliance rates decreasing to around those seen in the first cycle [24].

Future work could include creating an X-ray request template for traumatic ankle injuries; with the increasing use of Electronic Health Records, such a template could be built into systems to improve compliance. Regular departmental teaching could be implemented for Resident Doctors as part of postgraduate medical education, given that this cohort makes up a significant proportion of doctors working within the ED. Further assessment of local barriers to compliance, including knowledge and documentation quality, as well as the differences between hospitals within the same deanery, could help improve compliance at both the local and regional levels.

## Conclusions

This closed-loop Quality Improvement Project has demonstrated significant improvement in the ED documentation and X-ray request compliance with Ottawa Foot and Ankle Rules in a London DGH using a range of passive and active interventions, including teaching Resident Doctors. Future improvements could include a radiograph request template, assessing local barriers to compliance, and compliance rates between hospitals within the same deanery.
